# Small mosquitoes, large implications: crowding and starvation affects gene expression and nutrient accumulation in *Aedes aegypti*

**DOI:** 10.1186/s13071-015-0863-9

**Published:** 2015-04-28

**Authors:** David P Price, Faye D Schilkey, Alexander Ulanov, Immo A Hansen

**Affiliations:** Department of Biology, New Mexico State University, Las Cruces, NM USA; National Center for Genome Resources, Santa Fe, NM USA; Roy J. Carver Biotechnology Center, University of Illinois, Urbana-Champaign, Illinois, USA; Molecular Biology Program, New Mexico State University, Las Cruces, USA

**Keywords:** *Aedes aegypti*, fat body, RNAseq, Nutrition, Transcriptome, Metabolome, Immunity, Starvation

## Abstract

**Background:**

Environmental factors such as temperature, nutrient availability, and larval density determine the outcome of postembryonic development in mosquitoes. Suboptimal temperatures, crowding, and starvation during the larval phase reduce adult mosquito size, nutrient stores and affect vectorial capacity.

**Methods:**

In this study we compared adult female *Aedes aegypti*, Rockefeller strain, raised under standard laboratory conditions (Large) with those raised under crowded and nutritionally deprived conditions (Small). To compare the gene expression and nutritional state of the major energy storage and metabolic organ, the fat body, we performed transcriptomics using Illumina based RNA-seq and metabolomics using GC/MS on females before and 24 hours following blood feeding.

**Results:**

Analysis of fat body gene expression between the experimental groups revealed a large number of significantly differentially expressed genes. Transcripts related to immunity, reproduction, autophagy, several metabolic pathways; including amino acid degradation and metabolism; and membrane transport were differentially expressed. Metabolite profiling identified 60 metabolites within the fat body to be significantly affected between small and large mosquitoes, with the majority of detected free amino acids at a higher level in small mosquitoes compared to large.

**Conclusions:**

Gene expression and metabolites in the adult fat body reflect the individual post-embryonic developmental history of a mosquito larva. These changes affect nutritional storage and utilization, immunity, and reproduction. Therefore, it is apparent that changes in larval environment due to weather conditions, nutrition availability, vector control efforts, and other factors can affect adult vectorial capacity in the field.

**Electronic supplementary material:**

The online version of this article (doi:10.1186/s13071-015-0863-9) contains supplementary material, which is available to authorized users.

## Background

During the mid-twentieth century the distribution range of the yellow fever mosquito, *Aedes aegypti,* was greatly reduced by worldwide vector control programmes. *Ae. aegypti’s* comeback after these programmes were abandoned is responsible for the emergence of the most important arthropod-borne viral disease of our time, dengue fever. The WHO estimates that the dengue virus affects 50–100 million people a year with some estimates reaching 400 million per year, with 3.6 billion people currently at risk [1]. *Ae. aegypti* vectors a host of other diseases, including Yellow fever, which still affects some 200,000 people a year. The emerging Chikungunya virus, which infected more than 1.6 million people during one outbreak in India and Reunion island during 2005–2006, has just spread to the Caribbean and Florida [2-4]. All told, *Ae. aegypti* is, and will continue to be, of high medical significance for years to come.

A key organ for immunity, reproduction, hemolymph protein synthesis, metabolism, and nutrient storage in *Ae. aegypti* and insects in general is the fat body [5]. It is responsible for synthesizing a number of secreted proteins including: antimicrobial peptides such as defensins and cecropins, yolk proteins after a blood meal, and in larvae, hexamerin storage proteins [6-9]. Hexamerins and yolk proteins are secreted into the hemolymph for absorption by other tissues. In the case of yolk proteins, they are taken up by the ovaries for insertion into developing oocytes. In contrast, hexamerins are taken back into the larval fat body [5,7-9]. Fat body trophocytes are polyploid and contain a large number of ribosomes enabling rapid and massive production of such proteins [10].

As a key metabolic and storage organ, the fat body provisions large amounts of fats, carbohydrates and amino acids. These reserves are mobilized when needed: during flight, larval development, metamorphosis, and oogenesis. Yolk proteins are a way to transfer nutrient reserves to the developing oocytes. These proteins are highly expressed during the process of vitellogenesis, which takes place after a female mosquito takes a blood meal [11,12]. The fat body also participates in detoxification of xenobiotics and nitrogenous waste products which become highly elevated in adult female mosquitoes after blood meals [13].

The regulation of yolk protein precursors (YPP), or at the least of vitellogenin A, in anautogenous mosquitoes such as *Ae. aegypti* is accomplished with a triad of hormone signaling pathways and a nutrient signaling pathway- the target of rapamycin pathway (TOR) [14]. These regulation pathways keep vitellogenin A expression heavily repressed prior to the mosquito being competent for blood feeding and reproduction.

The juvenile hormone (JH) pathway is responsible in part for determining when the mosquito is competent for production of vitellogenins. It has been shown that nutritionally deprived mosquitoes have lower JH levels and do not as successfully develop eggs after one blood meal. However, treatment of small mosquitoes with JH can offset this [15].

Following a blood meal, when the mosquito has enough nutrients already stored, the ecdysone signaling pathway, insulin signaling pathway and TOR activate the expression of vitellogenin A [16-18]. Signals from the blood meal cause the brain to release ovary ecdysteroid hormone (OEH) into the hemolymph. Uptake of OEH into the fat body helps stimulate expression of yolk protein precursor genes. Insulin-like peptide signaling pathways appear to synergistically activate the expression of YPPs with OEH, through the activation of the forkhead box transcription factor [19,20].

In addition to these hormone signaling pathways, the TOR nutrient signaling pathway plays a major role in activation of YPP synthesis. Amino acids from the digested blood meal provide building blocks for the synthesis, but are also responsible for the activation of the TOR pathway in the fat body. The amino acids are taken up from the hemolymph by specific amino acid transporters into the fat body. This causes the activation, through an unknown mechanism, of the TOR signaling cascade. The end result of the activation of this pathway is activation of YPP synthesis via activated GATA transcription factor binding [9,21,22].

Variables in the larval environment, such as: temperature, population density, nutritional availability, and the presence of toxins have been shown to have significant effects upon time of larval development, adult body size, nutritional stores, immunity, reproductive capability and most significantly vector capacity [23-32]. In studies by Mitchell-Foster [23] and Alto [23], *Ae. aegypti* and *Culex pipiens* larvae raised under reduced nutrient conditions had decreased adult size, reduced numbers of eggs and smaller egg size [23,26]. Other studies found the amount of food larvae were given affects the time spent in the pupal life stage [23,24]. A study by Muturi [27] in *Ae. aegypti* investigated expression of immune transcripts and infection with sindbis virus under high/low temperature conditions and crowded/uncrowded conditions. It was found that high temperatures increased cecropin expression and higher larval density increased infection rates with sindbis virus at low but not at high temperatures. Studies by Dodson [28,29] found that nutritional stress and temperature increased larval development time and reduced adult size but there was no significant effect on vector competence for West Nile Virus. In a study by Sumanochitrapon [25] mosquitoes from several populations in Thailand were found to be less likely to become infected with dengue when raised under crowded, low nutrient conditions compared to low density, high nutrient conditions. However, the opposite was seen in two studies by Alto [30,31]. They found that smaller *Ae. aegypti* and *Aedes albopictus* have higher dengue virus infection and dissemination rates [30,31].

The aim of this study was to understand the effects of differing larval nutritional regimens on fat body gene expression and the metabolome in adult female mosquitoes. To this end, we generated two groups of mosquitoes, both from the same clutch of eggs of the highly inbred Rockefeller strain. We exposed the two groups to different regimes of population density and nutritional availability and produced well-nourished “large” and nutrient-deficient “small” mosquitoes. We conducted RNA-seq and metabolite profiling on the fat body of adult mosquitoes, pre- and post- blood meal raised under these different conditions and compared them in order to identify the molecular basis underlying these differences.

## Methods

### Mosquito culture

Mosquitoes were maintained as previously described [11], with the exception that they were fed as larvae solely on cat food (Special Kitty Original, Wal-Mart stores, Bentonville, AR). Large mosquitoes were generated by placing 100 larvae in 750 ml of deionized water in a 32 x 23 x 5 cm pan and fed ad libitum, typically a piece of cat food weighing ~0.2 g was kept in the pan at all times, and replaced when 1/2 to 2/3rd consumed. Small mosquitoes were generated by placing 750 larvae in the same type of pan with the same amount of water, but given 0.033+/−0.001 g of food on Monday, Wednesday and Friday. Pupae were collected from pans and placed into rearing cages to emerge. Cages were supplied with water and 20% sucrose solution.

### Mosquito size and weight measurement

Mosquitoes were frozen at −80°C overnight and weighed to record wet weight (19 large mosquitoes, 20 small mosquitoes). Mosquitoes were then placed at 50°C overnight and weighed again to record dry weight. Wing length was then recorded using a microscope slide with millimeter scale and dissecting microscope.

### Mosquito dissection & RNA-Seq

At 72 hours post-eclosion, mosquitoes from one cage were separated into two cages. One cage was blood-fed. At 24 hours post blood meal mosquitoes from both cages were dissected as previously described [11]. Abdominal preparations were dissected from *Aedes aegypti*, ROCK strain as described in Price 2011 [11]. Briefly, abdomens were dissected from whole mosquitoes, all internal organs were removed leaving an abdominal preparation consisting primarily of fat body tissue, with some epidermis, trachea and nervous tissue. Dissections occurred twice, once for each replicate. Approximately 11 fat bodies were dissected and pooled for each sample of small mosquito fat body and five into each sample of large fat body. Samples were stored at −80°C until all were collected and library preparation was ready to begin.

### Metabolomics

Mosquitoes were prepared and dissected as above, however, the abdomens were placed into ice cold PBS instead of trizol, then stored at −80°C until shipped for analysis on dry ice. Approximately 50 abdomens were pooled for each sample of large mosquitoes, and 70–80 for each sample of small mosquitoes in order to produce a total of ~50 mg of abdomens per sample.

Mosquito abdomens were pulverized in 1 mL of PBS buffer by ultrasound (5 x 1 min) with QSonica Microson XL2000 Ultrasonic Homogenizer (Qsonica, LLC, CT, USA), and subsequently extracted with 1 ml of 70% methanol and 1 ml of chloroform at room temperature. Every extraction was accompanied by centrifugation (5 min at 15000 g) and collection of the supernatants to the same tube. The final volume was evaporated under vacuum at −60°C and 5 μl of the Internal Standard (hentriacontanoic acid, 10 mg ml^−1^) was added prior to derivatization. Samples were derivatized and analyzed as described [33]. The instrument variability was 5%.

The spectra of all chromatogram peaks were compared with electron impact mass spectrum libraries NIST08 (NIST, MD, USA), W8N08 (Palisade Corporation, NY, USA), and a custom-built library of 520 unique metabolites. To allow comparison between samples, all data were normalized to the internal standard (IS) in each chromatogram and the sample weight. The spectra of all chromatogram peaks were evaluated using the AMDIS 2.69 (NIST, Gaithersburg, MD, USA) program. Metabolite concentrations are reported as relative concentration per gram dry weight (WW): N_i_ = X_i_ × X^−1^_IS_ × g WW^−1^.

### Illumina library preparation

Total RNA was extracted from these fat body samples according to the W.M. Keck Foundation protocol [34]. A Nanodrop 1000 (Thermo scientific) was used to quantify total RNA concentration. RNA quality was assessed visually using an RNA gel.

Four micrograms of total RNA (the recommended maximum) from each sample was used to prepare a library for each sample using the Illumina TruSeq RNA Sample Preparation Kit v2 according to the manufacturer’s protocol for low-throughput sample preparation, with modifications. The protocol was followed beginning with Purify and Fragment mRNA through Enrich DNA Fragments. Libraries were indexed separately for multiplexing.

Differences in the protocol and our preparation procedure were: using PCR strip tubes instead of PCR plates; the modification of step 26 in ligate adapters: Elute, Prime, Fragment mix was thawed on and mixed into each well of the RBP plate on ice rather than at room temperature. Ligation mix was thawed on ice and mixed into each well of the ALP plate on ice rather than at room temperature.

The resulting libraries were quantified using a Thermo Scientific Nanodrop 1000 and Agilent Bioanalyzer 2100, and then sent for sequencing. The sequencing laboratory further analyzed the libraries and pooled them for sequencing on a HiSeq2000, 1x100 bp reads.

### Bioinformatics

The obtained metabolite data matrix was divided onto 4 subgroups based on larval raising condition (small/large) and blood-fed/unblood-fed. All spurious metabolites derived from column bleed, reagent artifacts and xenobiotics were removed from the data sets. Missing values were inputted using half of the observed minimum positive detection value, assuming their level was below the instrument detection limit. Chemometric models were obtained with SIMCA P+ (12.0) programme (Umetrics, Umea, Sweden) using log-transformed and autoscaled data and validated by sevenfold Cross-Validation and permutation with 500 random. To address the problem of multiple comparisons the False Discovery Rate (FDR) test was used. The equipment has a sensitivity threshold below which it cannot detect the given metabolite. An average of 17 metabolites, which are part of those tested for a general panel, per sample were not detected.

Illumina reads were aligned to the *Ae. aegypti* reference transcripts (version 1.2 from Vectorbase) using bowtie [35,36]. Each library was aligned separately. Alignments in each library for each transcript were tallied from bowtie 2 results, then expression was compared between samples using the DESeq R package [37].

The Pearson correlation coefficient [38,39] was calculated between samples using the formula:

$$ r={\varSigma^{\mathrm{n}}}_{\mathrm{i}=1}\left(\left(\mathrm{Xi}-\overline{\mathrm{X}}\right)/\mathrm{S}\mathrm{x}\right)\left(\left(\mathrm{Y}\mathrm{i}\hbox{-} \mathrm{Y}\right)/\mathrm{S}\mathrm{y}\right) $$

For example, when comparing our small, not blood-fed treatment, X is repeat 1, Y is repeat 2. We measure the covariance of each transcript, and divide it by the standard deviation of each transcript, the product–moment. Summing the product–moment for each transcript is the correlation coefficient between the two repeats.

Gene ontology terms for all *Aedes aegypti* transcripts were retrieved with Biomart through Vectorbase [35,40]. Terms for differentially expressed transcripts were tested for enrichment using the R library GSEABase hyperGTest [41]. Terms were then counted for each transcript read, totaled, and parents drawn using the GO::Parser library for perl.

### Ethics statement

The research plan used for this work involving animals was specifically approved by the Institutional Animal Care and Use Committee (IACUC) at New Mexico State University under approval ID #2008-034. All procedures and care are described in the New Mexico State University Animal Care Facility Standard Operating Procedure and on file in the IACUC office there. All persons involved in animal work successfully completed Animal Welfare Training at New Mexico State University and were specifically trained in protocols used in the research plan. All New Mexico State University IACUC care and protocols follow the NIH guidelines described in Guide for the Care and Use of Laboratory Animals: Eighth Edition, ISBN-10: 0-309-15400-6.

## Results and Discussion

Our study was designed to identify differences in the metabolome and transcriptome of mosquitoes raised under different nutritional and crowding regimes, before and following blood feeding. The molecular changes we identified reflect alterations in immune, metabolic, reproductive transcripts and nutritional pathways between large and small mosquitoes. Alterations to these pathways are the molecular basis for differences in previous studies observing phenotypic changes in stressed mosquitoes - altered susceptibility to infection with pathogens such as dengue and reduced egg production in particular.

### The effect of diverse larval rearing conditions on wet and dry mass

The environment of the larval mosquito has a significant effect on the adult mosquito. Variables such as: temperature, population density, nutritional availability and toxin presence have been shown to have significant effects upon time of larval development, adult body size, nutritional stores, immunity, reproductive capability and most importantly, vector capacity [23-32,42].

Large and Small mosquitoes were raised as described in the Methods section. We found the average wet and dry body mass of female mosquitoes raised under uncrowded conditions (Large) to be significantly larger than the measurements of (Small) female mosquitoes raised under our crowded, starved conditions (see Table 1) (63% and 93% increase in mass respectively). This correlates with wing length, which has been used as a measure of mosquito size in previous experiments [23,25,42]. Based on wing length and body weight, we were able to successfully generate mosquitoes of two different average sizes from the same set of eggs.

**Table 1:** **Weight and wing length measurements of mosquitoes raised under standard laboratory conditions (large) vs. mosquitoes raised under crowded and nutrient deprived conditions, (small)**

**Measurement**	**Large**	**Std dev**	**Small**	**Std dev**	**p-value**
Mass (Wet), mg	1.89	0.18	1.16	0.11	0
Mass (Dry), mg	0.77	0.12	0.4	0.04	0
Wing Length (mm)	3.31	0.16	2.73	0.07	0

### RNA-seq

#### General RNA-seq results

**Figure 1:**
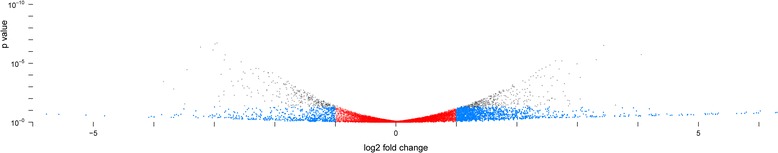
Volcano plot. Volcano plot generated with LBM vs. SBM transcript data. Transcripts altered at a less than log 2 fold level are in red, transcripts altered at a greater than two fold level, but with a p-value greater than 0.05 are in blue. Transcripts with an expression level altered more than log 2 fold and have a p-value less than 0.05 are black.

219 million 100 cycle reads were generated at NCGR for our four samples, Small No Blood meal (SNBF), Small Blood meal (SBM), Large No Blood meal (LNBF), Large Blood meal (LBM), a total of 21.9 gigabases of cDNA sequence. Of these, 184 million reads aligned to the reference *Ae. aegypti* transcriptome (84%) (version 1.2) [35]. To compare the similarity of our sample repeats, we analyzed transcript abundance between repeats using a Pearson correlation [38,39]. Coefficients generated between our sample repeats ranged between 0.987 (SBM1 and SBM2) and 0.997 (SNBF1 and SNBF2), signifying a high degree of positive correlation between our treatment repeats- very little alteration in transcript abundance between repeats. In contrast, comparing our blood-fed samples to non-blood-fed samples yielded correlations of 0.04 and 0.10, indicating large changes in the abundance of transcripts between the samples. Differential expression analysis identified 1496 transcripts with significantly altered expression (P < 0.05 by analysis with DESeq) between large and small groups following a blood meal (Additional file 1). 606 transcripts were found to be altered between large and small mosquitoes before a blood meal (Figure 1). These results indicate that there are a number of transcriptional differences between large and small mosquitoes, before and after blood meal. These differences likely reflect previously observed phenotype changes.

**Figure 2:**
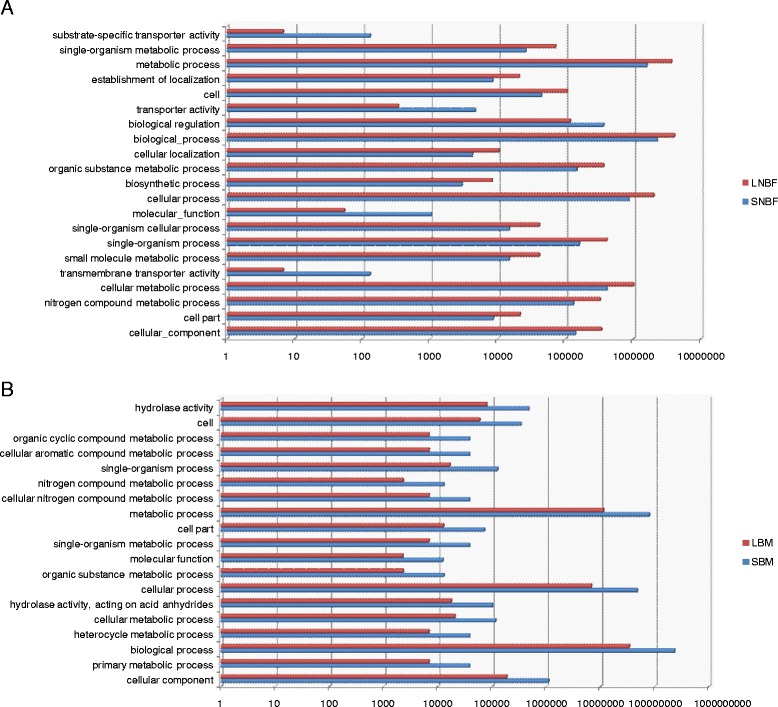
Numbers of GO terms found in differentially expressed transcripts from each sample, log base ten scale. **A**- small, not blood-fed (SNBF) vs large, not blood-fed (LNBF). **B**- large, blood-fed (LBM) vs small, blood-fed (SBM).

A gene ontology analysis of these differentially expressed transcripts is shown in Figure 2. Terms for membrane transport and biological regulation are more common in small mosquitoes vs large, prior to a blood meal. Several terms for metabolic processes are higher in large mosquitoes, including organic substance, small molecule and nitrogen compound metabolic processes. Following blood meal metabolic process terms dominate both samples and are more common in small mosquitoes. These results indicate that changes in metabolism between small and large mosquitoes make up a large portion of alterations in transcript expression. It is interesting that more metabolic GO terms are present in large mosquitoes prior to a blood meal, but following a blood meal, small mosquitoes increase the number of metabolic GO terms. This indicates that the small mosquitoes are utilizing the nutrients gained from the blood meal for their own metabolism instead of diverting them towards the production of eggs.

#### Vitellogenesis is impaired in small mosquitoes

The fat body stores large amounts of fats, carbohydrates and amino acids. These reserves are mobilized when needed, particularly during oogenesis. Yolk proteins are a way to transfer nutrient reserves from the fat body to the developing oocytes. These proteins are highly expressed during vitellogenesis, which takes place after a female mosquito takes a blood meal [11,12].

There were not any significant differences in reads between small and large mosquitoes (Table 2) associated with vitellogenesis (vitellogenins, vitellogenic cathepsin b (VCB) and vitellogenic carboxypeptidases (VCP)) prior to a blood meal, this is not unexpected as these genes are in a state of arrest prior to blood feeding.

**Table 2:** **Transcripts associated with vitellogenesis and cationic amino acid transport**

**Transcript ID**	**Description**	**Change**	**LBM**	**SBM**	**pval**				
AAEL010434-RA	vitellogenin-A	1.6	11913268.3	7453228	0.090				
AAEL006138-RA	vitellogenin-B	1.8	11378272.3	6201516	0.041				
AAEL006126-RA	vitellogenin-C	1.6	2609115.2	1664156	0.081				
AAEL006126-RB	vitellogenin-C	1.5	2938464.8	1905060	0.129				
AAEL012216-RA	VCB-A	2.0	362986.9	178678	0.019				
AAEL007585-RA	VCB-A	2.1	479528.7	226786	0.012				
AAEL015312-RA	VCB-A	1.3	820126.8	623994	0.254				
AAEL007599-RA	VCB-B	1.8	1208182.7	680214	0.045				
AAEL007590-RA	VCB-C	4.3	448.7	105	0.029				
AAEL009637-RA	VCB-D	2.6	72139.1	28123	0.000				
AAEL006563-RA	VCP-A	2.5	1475480.5	587557	0.000				
AAEL006542-RA	VCP-B	1.4	98665.1	71141	0.135				
**GeneID**	**Descr**	**change**	**LBM**	**SBM**	**pval**	**change**	**LNBF**	**SNBF**	**pval**
AAEL007818-RA	Trypsin 3A1 Precursor (EC 3.4.21.4)	2.98	708.9	238	0.000	0.00	136.1	40711	0.000
AAEL007818-RB	Trypsin 3A1 Precursor (EC 3.4.21.4)	2.80	495.1	177	0.000	0.00	100.8	29391	0.000
AAEL006425-RA	trypsin	0.06	91.0	1435	0.005	0.00	9.3	6041	0.000

Change- fold change, the number of reads in the large sample divided by the number of reads in the small sample. LBM, SBM, LNBF, SNBF- the number of reads in the respective sample aligning to the given transcript. Pval- the p-value calculated in DESeq.

Following a blood meal there were statistically significant changes in the number of reads across several classes of vitellogenesis related transcripts, including increases in transcript abundance in large mosquitoes for vitellogenin B (1.8 fold), VCB-A (2 fold), VCB-B (1.8 fold), VCB-C (4.3 fold) and VCP-A (2.5 fold), we observed a trend across all vitellogenesis related transcripts of fewer reads in small mosquitoes compared to large (Table 2). Reads were consistently 1.6-2.0 fold higher in large mosquitoes.

We did not find any of these vitellogenesis related genes to have altered expression levels pre-blood meal in small compared to large mosquitoes. Genes were not highly expressed in either sample.

While not statistically significant, we interpret this trend as a sign that the molecular process of vitellogenesis as a whole is being abbreviated in small mosquitoes- that blood meal derived energy and nutrients are siphoned away from egg production to general metabolic upkeep in small mosquitoes. Results of our KEGG metabolic pathway analysis provided additional evidence of this, specifically upregulation of oxidative phosphorylation, purine/pyrimidine metabolism and degradation/metabolism of certain amino acids (Additional file 2: Figure S1, Additional file 3: Figure S2, Additional file 4: Figure S3, Additional file 5: Figure S4, Additional file 6: Figure S5, Additional file 7: Figure S6, Additional file 8: Figure S7, Additional file 9: Figure S8 and Additional file 10: Figure S9).

Cationic amino acid Transporters (CATs) and Heterodimeric amino acid transporters (HATs), have a large effect on the process of vitellogenesis [43,44]. Previous studies have shown no significant upregulation of *Ae. aegypti* CAT (AaCAT) or AaHAT transporters at 24 hours following a blood meal, compared to immediately prior to blood feeding [44].

We did not find a significant difference between any SLC7 subgroup Cationic Amino acid Transporters (CATs), before or after a blood meal. However, several from the SLC7 Heterodimeric Amino acid Transporter subgroup (HATs) were significantly affected. HAT2, 3 and 4 were significantly upregulated in small mosquitoes prior to blood feeding (2.5 fold+). HAT1, 5 and 6 were significantly upregulated in small mosquitoes post blood feeding (5.25 fold+) (Table 2).

It has been demonstrated previously that the absence of cationic amino acids or knockdown of transporters of the SLC7 family, Cationic Amino acid Transporters (CATs) and Heterodimeric amino acid transporters (HATs), have a large effect on the process of vitellogenesis [43,44]. Previous studies have shown no significant upregulation of *Ae. aegypti* CAT (AaCAT) or AaHAT transporters at 24 hours following a blood meal, compared to immediately prior to blood feeding [44]. This makes the observed difference in expression level of AaHAT’s between small and large mosquitoes following a blood meal of particular interest. Results have indicated that the AaHAT family of amino acid transporters are vital in vitellogenesis as these transporters are able to affect the TOR signaling pathway by affecting phospho-S6 Kinase levels [44]. Additionally the *Drosophila* homolog of AaHAT2, minidiscs, plays a role in larval development of imaginal disks. It has been shown that minidiscs mediated transport, presumably in the fat body, affects imaginal disk growth [45]. Based upon these results we hypothesize that these transporters are being expressed at higher levels in the fat body of small mosquitoes in order to complete a facet of development or nutrient storage in the adult mosquito, likely by affecting the TOR signaling pathway [42].

##### Autophagy-related genes

Programmed autophagy is a vital part of the vitellogenic process, particularly its proper termination. Several autophagy related transcripts, ATG1, ATG6 and ATG8, have previously been shown to be upregulated maximally in the fat body by or at 36 hours post blood meal [46].

Prior to blood feeding the autophagy pathway was not affected (Additional file 11: Figure S10, KEGG autophagy pathway, NBF), though ATG7, 8 and 13 were upregulated slightly in small mosquitoes. However, many free amino acid levels (Table 3) were higher in non-blood-fed small mosquitoes vs. large mosquitoes. Trypsin 3A1 Precursor (AAEL007818) and Trypsin (AAEL006425) transcript levels were also found to be higher in small mosquitoes before a blood meal.

**Table 3:** **Levels of free amino acids detected in our metabolomic analyses**

**Compound**	**SBM v LBM**	**t-val**	**q-val**	**SNBM v LNBM**	**t-val**	**q-val**		
Alanine	144.3	344.5	0.024	0.036	135.1	85.4	0.023	0.036
Aspartic acid	3.9	1.2	0.005	0.036	11.0	0.2	0.001	0.036
Glutamic acid	76.2	69.0	0.613	0.294	22.9	4.4	0.366	0.206
Glycine	252.8	229.2	0.683	0.316	122.2	15.4	0.003	0.036
Histidine	5.0	0.2	0.003	0.036	0.2	0.2	ND	ND
Isoleucine	27.1	115.4	0.006	0.036	20.0	6.6	0.127	0.083
leucine	36.2	79.6	0.101	0.075	24.7	5.7	0.002	0.036
Lysine	22.4	0.2	0.002	0.036	48.5	0.2	0.000	0.036
Methionine	12.5	8.1	0.134	0.083	9.3	0.2	0.012	0.036
Phenylalanine	46.8	42.2	0.813	0.351	31.9	0.2	0.003	0.036
Proline	356.5	962.4	0.174	0.103	33.7	10.2	0.018	0.036
Serine	79.3	196.4	0.133	0.083	62.0	49.7	0.812	0.351
Threonine	64.4	215.9	0.002	0.036	43.6	34.0	0.392	0.211
Tryptophan	38.7	20.6	0.045	0.044	0.2	0.2	ND	ND
Tyrosine	242.6	289.7	0.546	0.272	13.7	54.6	0.001	0.036
Valine	44.8	64.2	0.413	0.214	45.3	16.2	0.021	0.036

Leu, Lys, Phe, Val, Ala, Asp, Glu, Gly and Pro levels were found to be higher in small mosquitoes prior to blood meal, while Tyr was found to be higher in large mosquitoes. Post blood meal Ile, Thr, Trp, and Ala were found to be higher in large mosquitoes while Lys, Asp and His were found to be higher in small mosquitoes.

Following a blood meal, genes of the autophagy pathway (Additional file 12: Figure S11, KEGG autophagy pathway BM), were found to be significantly down regulated in small mosquitoes. This lends further evidence that vitellogenesis is altered in small mosquitoes.

#### The metabolome and metabolic pathways Are altered between small and large mosquitoes

The fat body plays several important roles but is primarily described as an organ for storage and release of energy and nutrients [5]. To explore this role we performed an analysis of the fat body metabolic pathways by mapping our transcripts to the KEGG pathways for *Aedes aegypti* to identify differences in active metabolic pathways [47]. Due to the importance of the fat body in storing and releasing nutrients, we performed a metabolomic analysis using mass spectrometry and liquid chromatography to identify a panel of metabolites. We used this panel of metabolites to identify differences in nutritional storage between the fat body of large and small mosquitoes.

**Figure 3:**
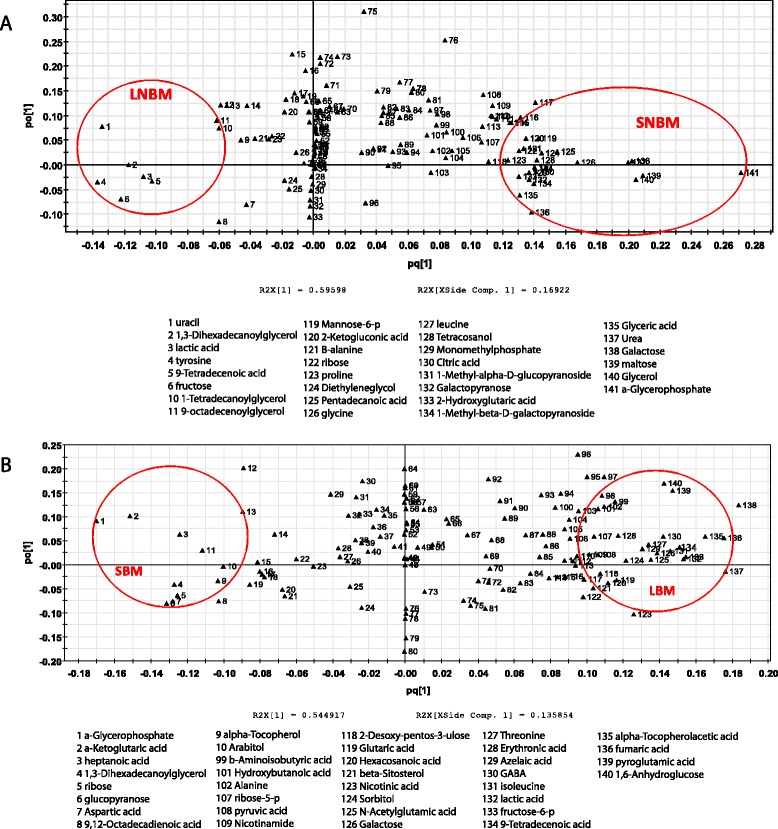
Putative Metabolite Biomarkers. **A**. Circled metabolites were identified as putative biomarkers using OPLS-DA analysis for large mosquitoes, prior to a blood meal or small mosquitoes prior to a blood meal. **B**. Circled metabolites were identified as putative biomarkers using OPLS-DA analysis for large mosquitoes, following a blood meal or small mosquitoes following a blood meal.

We obtained relative concentration results for 138 metabolites. There were 58 significant changes between SBM vs. LBM and 65 significant changes between SNBM vs. LNBM. From these affected metabolites, we selected outliers, based on OPLS-DA analysis, as putative biomarkers (q ≤ 0.03) for our different nutritional/size and blood feeding states (Figure 3). Between LBM and SBM 35 metabolites were selected, between LNBM and SNBM 36 metabolites were selected (Additional file 13).

Prior to a blood meal, 3 pathways were down-regulated (oxidative phosphorylation, N-Glycan biosynthesis and lysine degradation) in small mosquitoes, compared to large mosquitoes (p < 0.05). Following blood meal, 11 KEGG pathways were up-regulated in small mosquitoes (P < 0.05) following a blood meal (Table 4). Significant changes were observed in alpha-tocopherol levels, increased in small vs. large (21.5 vs. 8.1, t = 0.048, q = 0.021) before and after (17.0 vs. 7.7, t = 0.037, q = 0.023) a blood meal.

**Table 4:** **A. KEGG pathways altered between Large and Small, prior to blood feeding; B. KEGG pathways altered between Large and Small, post blood feeding**

**KEGG id**	**Name**	**p-value**
**A. KEGG pathways altered between large and small, pre-bloodmeal**		
aag00190	Oxidative phosphorylation	0.04
aag00510	N-Glycan biosynthesis	0.01
aag00310	Lysine degradation	0.04
**B. KEGG pathways altered between large and small, post-bloodmeal**		
aag00190	Oxidative phosphorylation	1.68E-11
aag00230	Purine metabolism	2.68E-05
aag00280	Valine, leucine and isoleucine degradation	7.79E-04
aag00240	Pyrimidine metabolism	5.30E-03
aag00260	Glycine, serine and threonine metabolism	1.59E-02
aag00640	Propanoate metabolism	2.84E-02
aag00250	Alanine, aspartate and glutamate metabolism	3.03E-02
aag00630	Glyoxylate and dicarboxylate metabolism	3.04E-02
aag00310	Lysine degradation	3.17E-02
aag00563	Glycosylphosphatidylinositol(GPI)-anchor biosynthesis	3.27E-02
aag00620	Pyruvate metabolism	3.77E-02

Alpha-tocopherol, a form of vitamin E, is necessary for development of many insect species and dietary supplementation has been reported to have an effect on longevity, development and fecundity, however, effects on longevity and fecundity reported have been inconsistent in insects [48-50]. Higher levels of supplementation have a protective effect against oxidative stress [50]. We hypothesize that in the case of small mosquitoes, increased levels of alpha-tocopherol may be serving to protect from stresses associated with starvation through antioxidant activity.

There were significantly increased amounts (by t value) of 9/17 free amino acids in small mosquitoes prior to a blood meal (A, D, G, L, M, F, P, Y, V), and increased levels of five amino acids in small mosquitoes following a blood meal (A, D, H, I, K, T) (Table 3). In addition, there were significantly higher levels of glycerol in the fat bodies of small mosquitoes than large mosquitoes, prior to blood feeding (3493.4 vs. 170.2, p = 7.5E-5, q = 0.008).

The trend of increased levels of free amino acids (Table 3)- (9 of the 17 reported significantly changed, two not detected, 4 non-significantly up, the only significantly down being tyrosine) and trypsin (16 fold increase) transcripts (Table 2) is a sign of autophagy occurring in small mosquitoes, prior to blood feeding [51,52]. Some autophagic mechanism may be responsible for elevated amino acid and trypsin transcript levels in the adult, or the small mosquitoes may have been undergoing autophagy during the larval and pupal stages.

The elevated levels of glycerol observed in small mosquitoes prior to blood feeding may indicate an increased level of breakdown of triglycerides for energy compared to large mosquitoes, indicating a shift toward energy store breakdown vs. accumulation. This may have implications for vitellogenesis.

Following a blood meal, free amino acid patterns in the fat body change. Only lysine, tryptophan, aspartic acid and histidine were found to be in significantly higher concentrations in small mosquitoes, while isoleucine, threonine and alanine were found to be in significantly higher concentrations in large mosquitoes. In addition, KEGG pathways for the degradation or metabolism of amino acids were significantly up-regulated in small mosquitoes after a blood meal (Table 4). These results indicate that small mosquitoes are using blood meal-derived amino acids and previously free amino acids in metabolic processes other than vitellogenesis [17]. We found transcripts responsible for converting alanine to pyruvate and pyruvate to products such as oxaloacetate and lactate to be more highly expressed in small mosquitoes following a blood meal, indicating amino acids are being used as an energy source rather than as protein building blocks (Additional file 2: Figure S1 Ala_Asp_Glu_BM).

An upregulation of the KEGG pathway for oxidative phosphorylation in large mosquitoes (Additional file 5: Figure S4) indicates that prior to blood feeding small mosquitoes are producing ATP and NADH at a lower level, then shift to producing these energy products following the influx of blood meal derived energy and nutrients. These results fit with the idea that energy derived from the blood meal in small mosquitoes is shifted towards replenishing the mosquitoes own stores, rather than being used for reproduction.

#### Juvenile hormone and ecdysone breakdown is altered in small mosquitoes

##### Insect hormone biosynthesis

The metabolism of ecdysone and juvenile hormone (JH) are extremely important in mosquito development and reproduction. The KEGG pathway consists of two relatively unrelated pathways evaluated together, and certain individual enzymes in these pathways have been suggested to be rate limiting- a change in the expression of these genes alone may have a large effect on amounts of JH and ecdysone produced by a tissue [53].

However, in insects the site of synthesis of JH is the corpora allata while pro-thoracic gland and ovaries synthesize ecdysone. We did not expect the bulk of enzymes in the pathways for synthesis to be expressed in the fat body, rather just the enzymes responsible for the breakdown of JH, and the breakdown or conversion of ecdysone to 20-ecdysone.

We did not find the KEGG pathway for insect hormone biosynthesis as a whole to be significantly changed between small and large mosquitoes, before or after a blood meal. Before a blood meal in the pathway for the synthesis of juvenile hormone (JH), we found there was a slight decrease in the breakdown step for JHIII to JHIII diol in small mosquitoes, catalyzed by juvenile hormone epoxide hydrolase. In the pathway for ecdysone, we found a slight decrease in the step leading to 20-hydroxyecdysone, and an increase in the step leading to the breakdown of 20-hydroxyecdysone to 20,26 dihydroxyecdysone. Following a blood meal, we found no differences in the juvenile hormone pathway. We did find decreases in small mosquitoes in the steps leading to 20-hydroxyecdysone and its breakdown.

Changes to individual transcripts prior to a blood meal (Additional file 14: Figure S12) in the KEGG pathway for insect hormone biosynthesis could indicate that juvenile hormone is converted to JH III diol, keeping JH levels higher in small mosquitoes. This may reflect an extended period of time to complete sexual maturity for the small mosquitoes, as a decrease in JHIII levels accompany maturation [54]. Higher JH levels have also been tied to reabsorption of nutrients from follicles when under nutritional stress [55]. Reduced conversion of ecdysone to 20-hydroxyecdysone is expected in small mosquitoes following a blood meal, and the corresponding reductions in yolk protein transcripts we observed are likely at least in part due to this alteration [8].

#### Small mosquitoes have altered immunity

Several immune pathways exist within mosquitoes to deal with pathogens and keep infection to a minimum. The JAK/STAT, Toll/IMD, RNA interference and apoptotic pathways all contribute to preventing and clearing infections [56-59]. RNA interference plays a key role in immunity against viruses, which generate double stranded RNA, including dengue [60,61]. Apoptotic genes have also been shown to modulate dengue infection [62].

##### Apoptosis

AeIAP1 (Inhibitor of Apoptosis) levels have been shown to be altered between strains of *Ae. aegypti* susceptible and refractory to dengue. Knockdown of genes whose active protein form is regulated by AeIAP1 has been shown to increase dengue susceptibility [62]. AeIAP1 (inhibitor of apoptosis) was more highly expressed in small mosquitoes compared to large, before (5866 vs. 6033 reads, p = 0.032) and after (3365 vs. 8661 reads, p = 0.036) a blood meal. These results weakly indicate that small mosquitoes may be able to more effectively respond to dengue infection by clearing infected cells.

##### JAK-STAT pathway

Canonically, JAK/STAT has been associated with the immune responses against virus infection and has been shown to affect dengue infection, but has been shown to respond to fungi [56,57]. The JAK-stat pathway was unchanged with the exception of DRVF2, which was upregulated in small mosquitoes prior to a blood meal. However, its expression level was low in both samples.

##### TOLL-IMD Pathways

The Toll pathway has also been shown to respond to infection with dengue and other viruses, as well as to certain types of bacteria and fungi, while IMD is thought to respond to gram negative bacteria primarily [58,59].

Following a blood meal we found spaetzle 3 A & B (4 fold), several cecropins (AAEL000625 & AAEL000621 11+ fold) and defensin (7.14 fold) to be upregulated in small mosquitoes following a blood meal. ClipB26 was found to be upregulated (2.6 fold) following a blood meal in large mosquitoes, along with slight upregulation of diptericin and PGRPS1 (1.1 fold).

In small mosquitoes, pre-blood meal, a series of cecropins (AAEL000611, AAEL015515, AAEL000621) were upregulated (30 fold), as well as Peptidoglycan recognition protein LB (PGRPLB) (30 fold). Additionally spaetzle 2 and Cactus were upregulated (Table 5) (6.6 fold and 20 fold, respectively).

**Table 5:** **Altered immune transcripts**

**GeneID**	**Descr**	**LBM/SBM**	**LBM**	**SBM**	**pval**				
AAEL017555-RA	Clip-domain serine protease, family B	2.62	152	58	0				
AAEL003832-RA	Defensin-C Precursor	0.14	43.7	322	0				
AAEL014950-RA	Spaetzle-likecytokine.	0.13	42.8	317	0				
AAEL000625-RA	Cecropin,Anti-Microbial Peptide.	0.04	1.8	51	0.01				
AAEL000621-RA	Cecropin,Anti-Microbial Peptide.	0.09	7.3	81	0.01				
AAEL008596-RA	Spaetzle-likecytokine. Spaetzle 2	0.15	33.7	226	0.02				
AAEL006936-RB	conserved hypothetical protein	1.14	369.5	323	0.02				
AAEL009474-RA	PeptidoglycanRecognition Protein (Short)	1.09	666.2	609	0.03				
AAEL004833-RA	Diptericin,Anti-Microbial Peptide.	1.16	100.1	86	0.04				
**GeneID**	**Descr**	**lnbf/snbf**	**LNBF**	**SNBF**	**pval**				
AAEL000625-RA	Cecropin,Anti-Microbial Peptide.	0.02	5.4	216	0				
AAEL000621-RA	Cecropin,Anti-Microbial Peptide.	0.02	13.7	555	0				
AAEL000896-RA	conserved hypothetical protein (DRVF2)	0.08	16.2	204	0				
AAEL000611-RA	Cecropin, Anti-Microbial Peptide.	0.03	28.9	1090	0				
AAEL000709-RB	TOLLpathway signalling.	0.31	1959	6338	0.01				
AAEL010171-RA	PeptidoglycanRecognition Protein (Long)	0.2	60.7	306	0.01				
AAEL015515-RA	Cecropin,Anti-Microbial Peptide.	0.02	1	55	0.01				
AAEL000627-RA	Cecropin-A Precursor	0.04	2.4	57	0.02				
AAEL001435-RA	Spaetzle-likecytokine.	0.24	29.9	123	0.04				
AAEL000598-RA	Cecropin,Anti-Microbial Peptide.	0.05	2.4	46	0.04				
AAEL009074-RA	AeIAP1	0.97	5866.4	6033	0.032	0.39	3365.8	8661	0.036
ORL Strain									
**GeneID**	**Descr**	**LBM/SBM**	**LBM**	**SBM**	**pval**	**lnbf/snbf**	**LNBF**	**SNBF**	**pval**
AAEL000611-RA	Cecropin, Anti-Microbial Peptide.	0.31	11.8	38	0.535	0.03	28.9	1090	0
AAEL015515-RA	Cecropin,Anti-Microbial Peptide.	0.36	1.8	5	1.000	0.02	1	55	0.01
AAEL000621-RA	Cecropin,Anti-Microbial Peptide.	0.09	7.3	81	0.01	0.02	13.7	555	0
AAEL014382-RA	C-Type lectin (CTL) - mannose binding.	1.91	1691.8	885	0.000	0.59	264.3	447	0.436
AAEL005431-RA	Clip-domain serine protease, family B.	0.74	1227.7	1650	0.332	0.36	671.6	1843	0.087
AAEL004758-RA	pupal cuticle protein, putative	0.31	87.4	284	0.314	1.09	189.4	174	0.551
BKK strain									
AAEL007585-RA	cathepsin b	2.11	479528.7	226786	0.012	1.12	209.5	187	0.657
AAEL012216-RA	cathepsin b	2.03	362986.9	178678	0.019	0.99	149.3	151	0.817
AAEL015312-RA	cathepsin b	1.31	820126.8	623994	0.254	1.14	371.0	326	0.797
AAEL009642-RA	cathepsin b	1.51	74103.0	48932	0.045	0.74	384.3	522	0.835
AAEL013417-RA	fibrinogen and fibronectin	0.30	2380.8	7960	0.340	0.59	361.7	614	0.556
AAEL000726-RA	fibrinogen and fibronectin	0.23	1.8	8	0.886	1.47	1.5	1	1.000
AAEL008646-RA	fibrinogen and fibronectin	1.52	1256.8	829	0.016	1.04	1274.6	1224	0.671
AAEL013498-RA	Prophenoloxidase	0.51	1698.2	3304	0.994	1.41	831.7	588	0.656
AAEL015116-RA	prophenoloxidase	0.68	1953.9	2865	0.658	1.32	776.8	588	0.612
AAEL014755-RA	tep2	0.73	3007.8	4141	0.209	0.46	6831.9	14889	0.104

The increase in transcript abundance of cecropins in small mosquitoes, pre-blood meal were similar to those observed as basal changes in expression in the dengue refractory Orl strain compared to Rock, known as a dengue susceptible strain [63]. However, the C-type lectin and clip domain serine protease found to be affected in the Orl strain were not significantly affected in our sampling.

Molecular variations of this nature downstream of nutrition or other stresses are key to differences in vectorial capacity. We speculate that these observed changes in this may lead to these particular small mosquitoes being refractory to dengue compared to our large mosquitoes and help explain variations in vector capacity observed between small and large mosquitoes [25,27-31]. However, it has to be stressed that our study used the highly inbred Rockefeller strain and that the outcome of starvation stress on other strains of *Ae. aegypti* might translate to different effects on vectorial capacity.

## Conclusions

Significant alterations to the metabolome, metabolic pathways, immune pathways, and vitellogenic related transcripts between small and large mosquitoes reflect significant changes in fecundity and vectorial capacity between these groups of mosquitoes.

Our results correlate well with previous work showing lowered or no egg production after the first blood meal in mosquitoes starved and/or crowded as larvae [23,26]. The results of lowered vitellogenesis-related transcripts were expected. However, the metabolic and transcriptional basis for these changes is of interest. In large mosquitoes, nutrients from the first blood meal are allowing the process of vitellogenesis to occur. In contrast, in small mosquitoes many of these nutrients, such as reduced levels of alanine and upregulation of pathways turning alanine into pyruvate and other energy substrates, are being diverted to fill in the nutritional deficits left from starvation conditions they experienced as larvae.

We have observed a possible molecular basis for nutritional stress causing changes in susceptibility to disease. Upregulation of several cecropin transcripts in small mosquitoes in a pattern similar to changes previously observed between strains of *Aedes aegypti* susceptible (ROCK) and refractory (Orl) to dengue infection leads us to hypothesize that ROCK strain *Ae. aegypti* raised under nutritional stress may be refractory to dengue infection compared to those raised under standard conditions. This does not necessarily mean smaller mosquitoes are “safer”- the combination of crowding and food reduction means these mosquitoes require more blood meals to produce eggs and may bite more often.

It is possible, and probable, that different strains of *Ae. aegypti* may respond differently to these stresses- what increases immunity in one strain may decrease it in another strain. We also do not expect different stresses- temperature, insecticides, or predation, to produce the same results. However, these observed changes are important considerations when working to control populations of this globally important vector. In addition, our results stress the importance of raising *Ae. aegypti* under standardized conditions for comparative molecular and immunological studies because deviations from standardized laboratory conditions may have a very large impact on gene expression, reproductive physiology, and immunity.

## Additional files

Additional file 1:
**Sequencing alignment and DESeq statistical results.**
Additional file 2: Figure S1. Annotated KEGG map for alanine, asp, glu, following BM. Additional file 3: Figure S2. Annotated KEGG map for gly, ser, thr, following BM. Additional file 4: Figure S3 Annotated KEGG map for lysine following BM. Additional file 5: Figure S4. Annotated KEGG map for oxidative phosphorylation following BM. Additional file 6: Figure S5. Annotated KEGG map for purine metabolism following BM. Additional file 7: Figure S6. Annotated KEGG map for pyrimidine metabolism following BM. Additional file 8: Figure S7. Annotated KEGG map for pyruvate following BM. Additional file 9: Figure S8. Annotated KEGG map for leu, val, ile following BM. Additional file 10: Figure S9 Annotated KEGG map for oxidative phosphorylation, NBF. Additional file 11: Figure S10. Annotated KEGG map for Autophagy, NBF. Additional file 12: Figure S11. Annotated KEGG map for Autophagy, following BM. Additional file 13:
**Additional figure legend for Figure** 3 **.**
Additional file 14: Figure S12. Annotated KEGG map for insect hormone biosynthesis, NBF.

## Abbreviations

LNBF: Large not blood-fed
LBM: Large post blood meal
SNBF: Small not blood-fed
SBM: Small post blood meal
VCB: vitellogenic cathepsin b
VCP: vitellogenic carboxypeptidases
SLC7: Solute carrier family 7
CAT: Cationic amino acid Transporter
HAT: Heterodimeric amino acid transporter
JH: Juvenile hormone
PGRPLB: Peptidoglycan recognition protein LB
PGRPS1: Peptidoglycan recognition protein S1
