# Supplementary figures and images for: Small mosquitoes, large implications: crowding and starvation affects gene expression and nutrient accumulation in Aedes aegypti
Source: Parasit Vectors. 2015 Apr 28;8:252. doi: 10.1186/s13071-015-0863-9 (PMC4415286; doi:10.1186/s13071-015-0863-9)

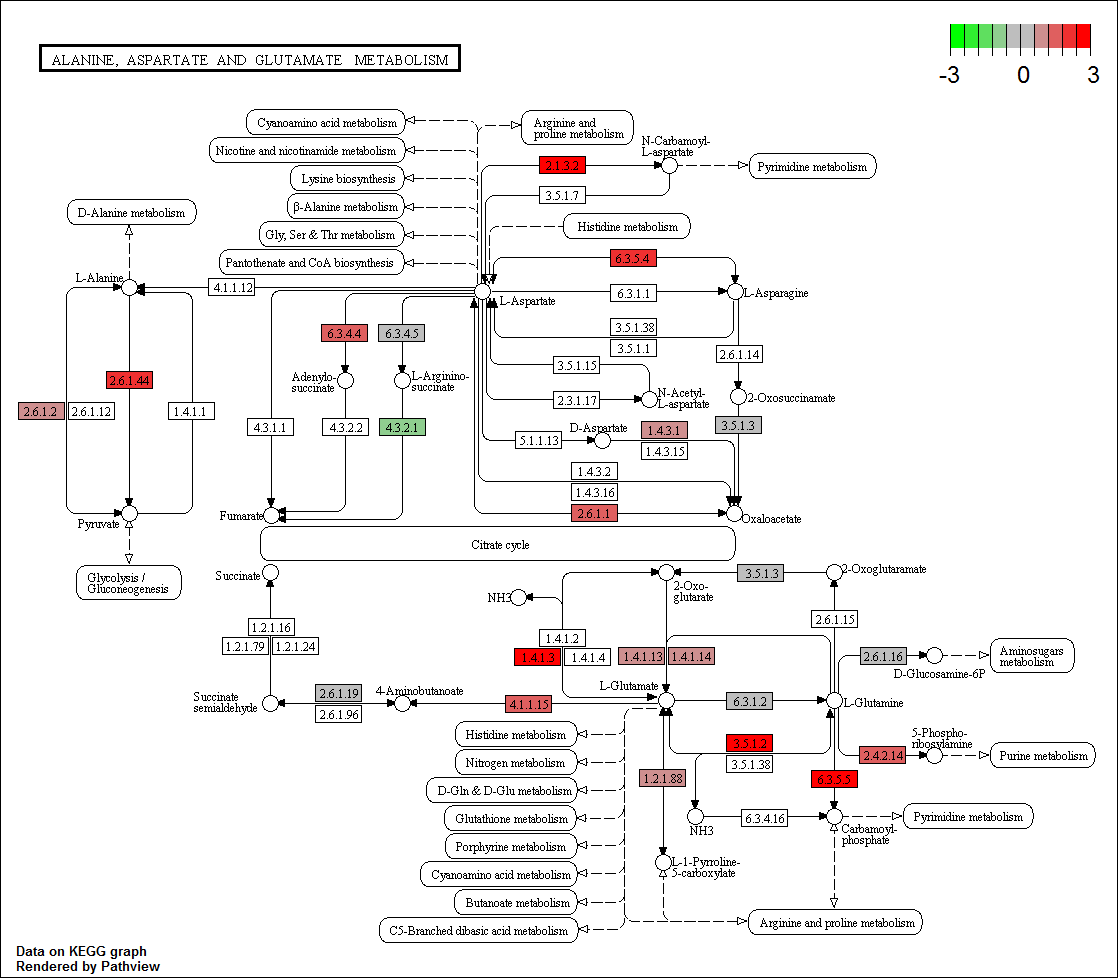

Supplement: Additional file 2: Figure S1. — Annotated KEGG map for alanine, asp, glu, following BM. [file 13071_2015_863_MOESM2_ESM.png]

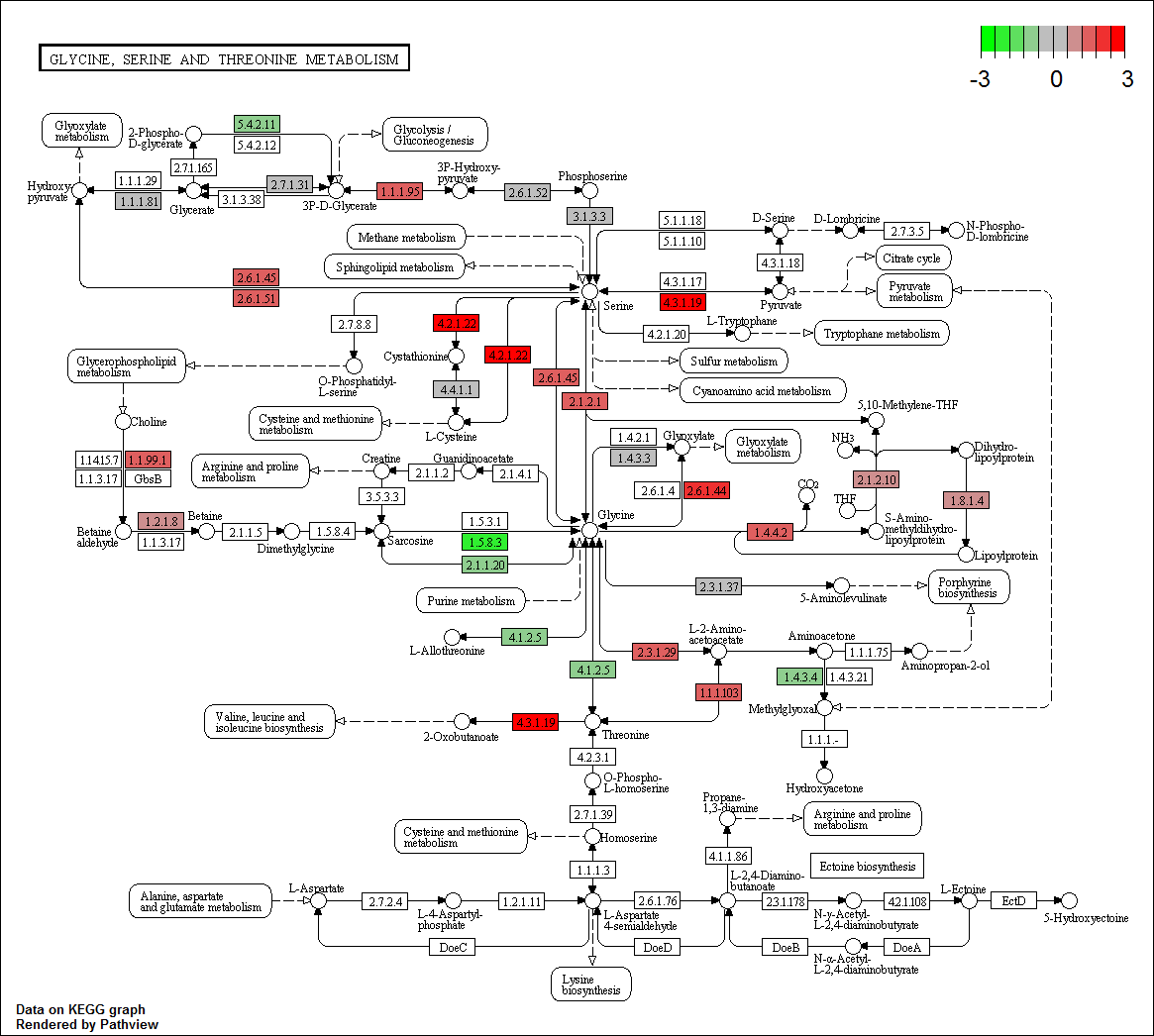

Supplement: Additional file 3: Figure S2. — Annotated KEGG map for gly, ser, thr, following BM. [file 13071_2015_863_MOESM3_ESM.png]

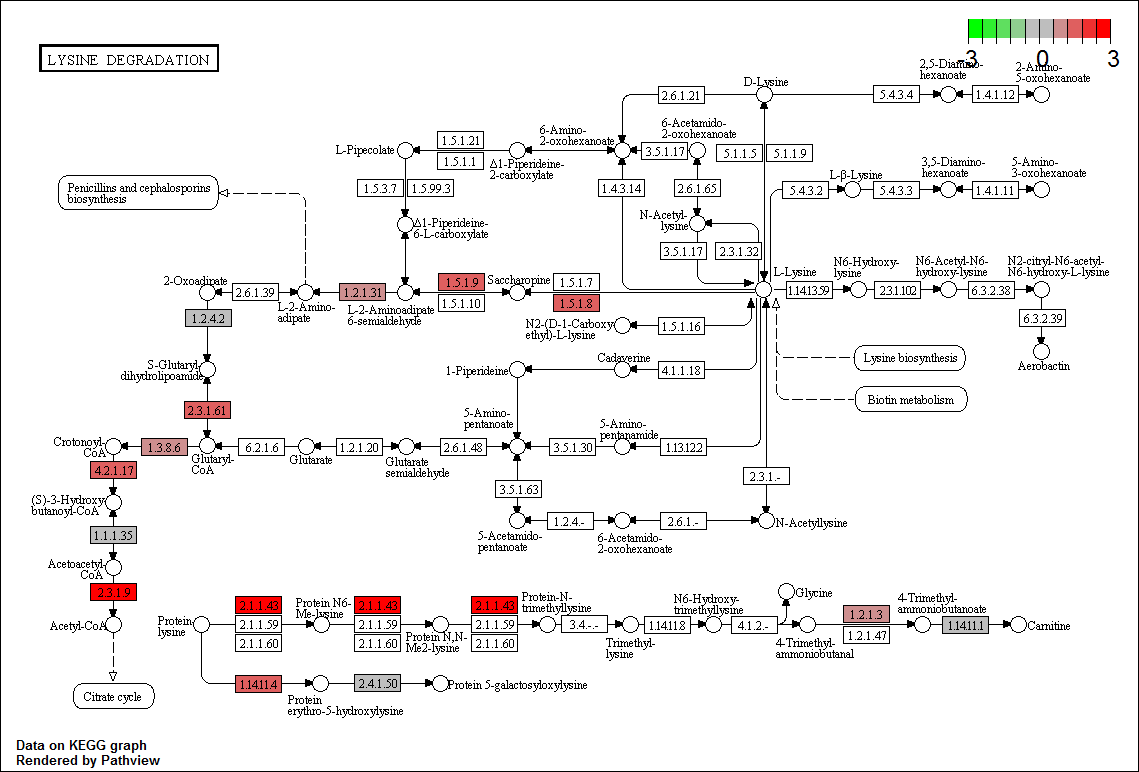

Supplement: Additional file 4: Figure S3 — Annotated KEGG map for lysine following BM. [file 13071_2015_863_MOESM4_ESM.png]

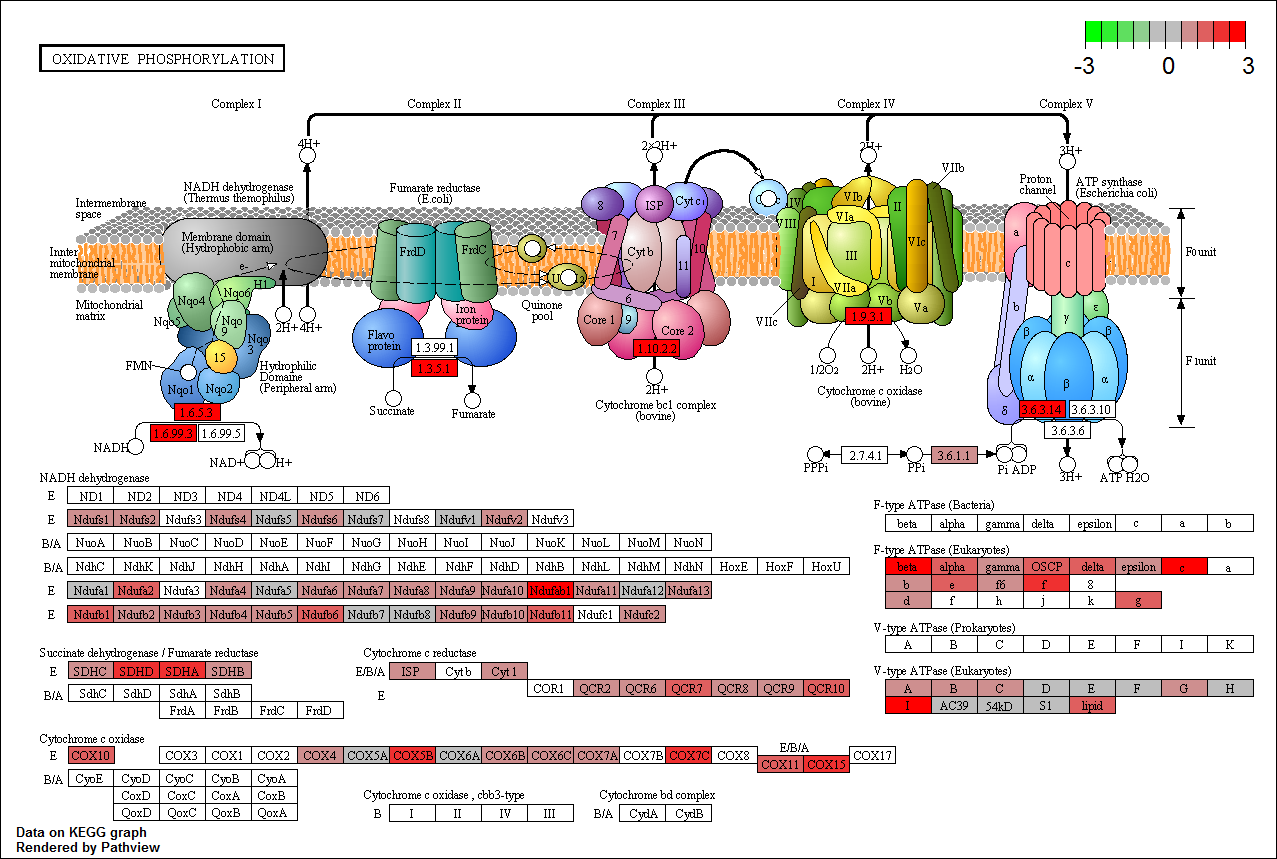

Supplement: Additional file 5: Figure S4. — Annotated KEGG map for oxidative phosphorylation following BM. [file 13071_2015_863_MOESM5_ESM.png]

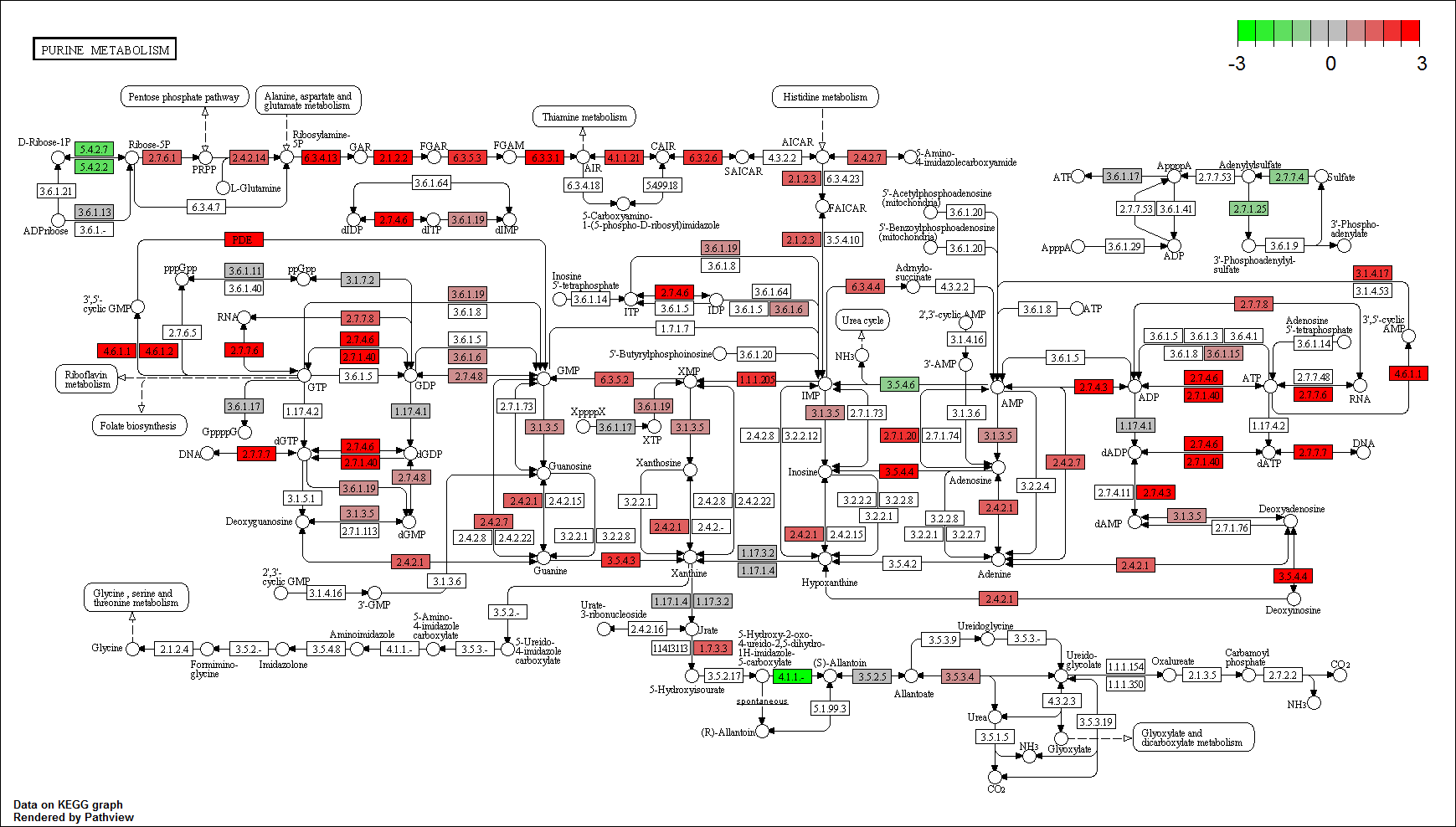

Supplement: Additional file 6: Figure S5. — Annotated KEGG map for purine metabolism following BM. [file 13071_2015_863_MOESM6_ESM.png]

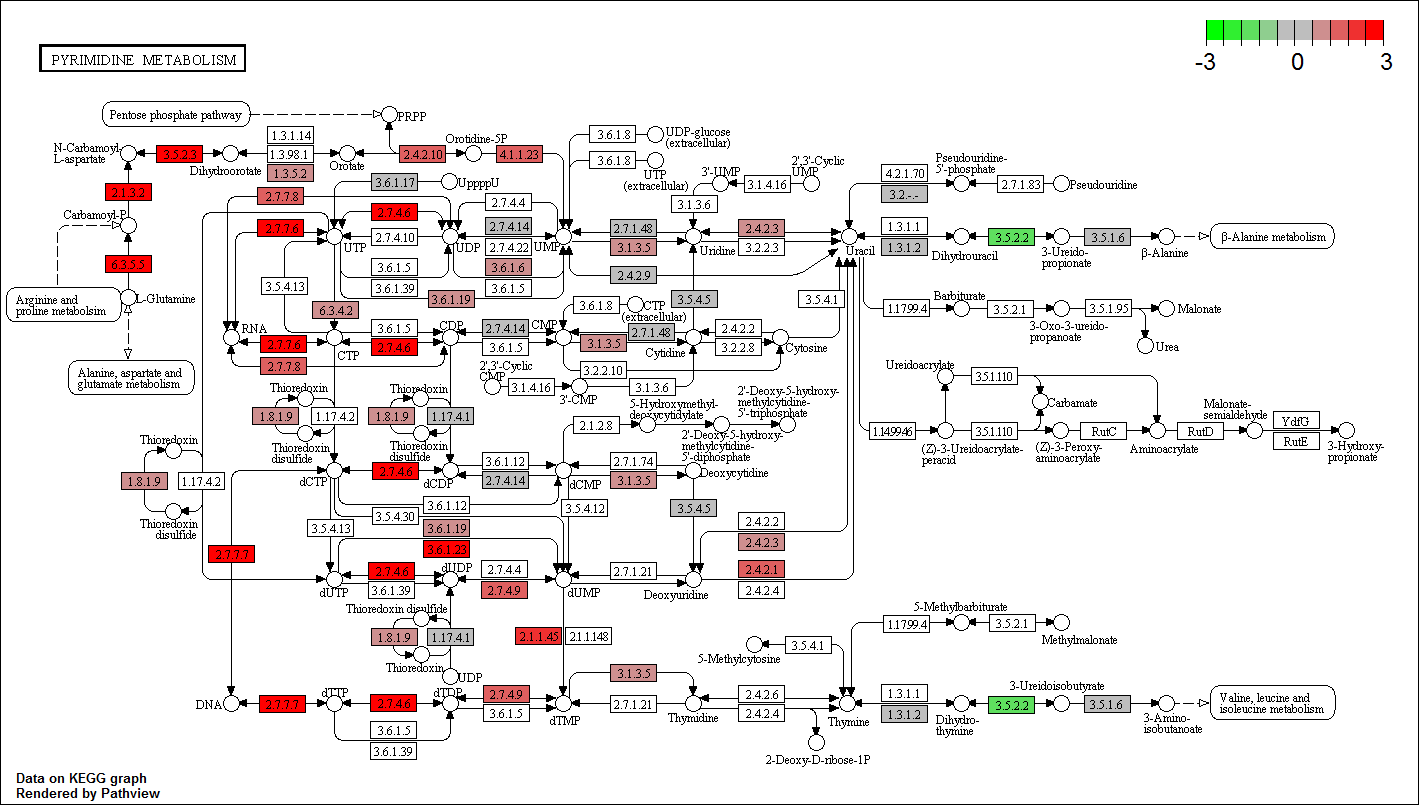

Supplement: Additional file 7: Figure S6. — Annotated KEGG map for pyrimidine metabolism following BM. [file 13071_2015_863_MOESM7_ESM.png]

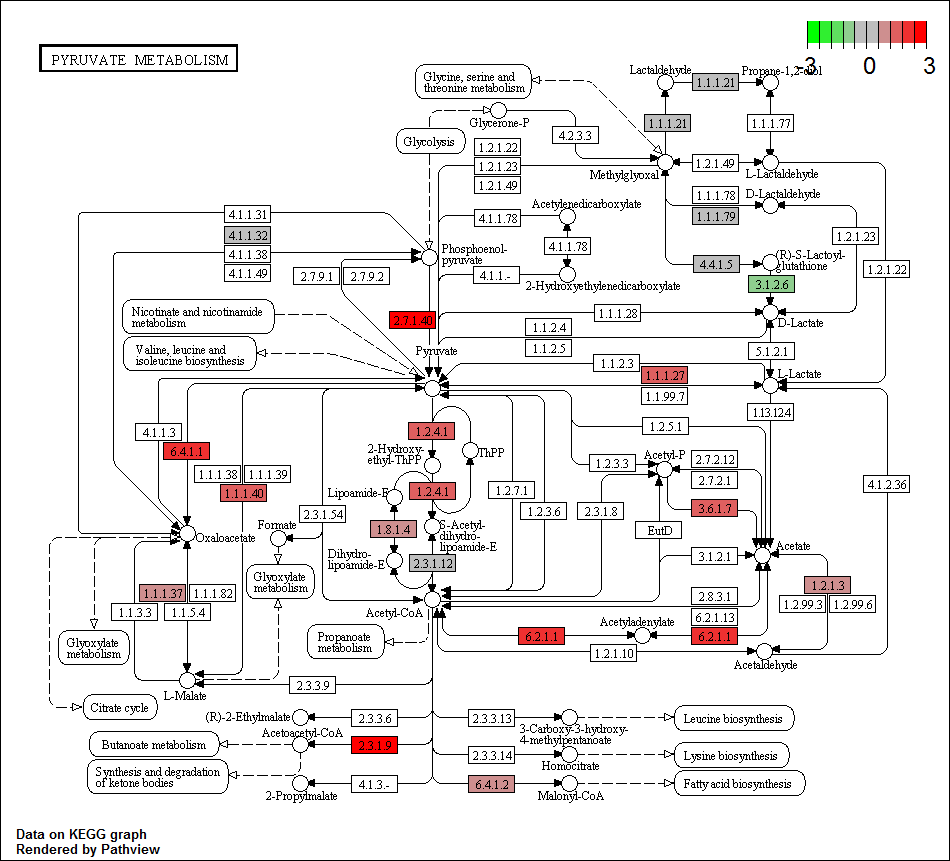

Supplement: Additional file 8: Figure S7. — Annotated KEGG map for pyruvate following BM. [file 13071_2015_863_MOESM8_ESM.png]

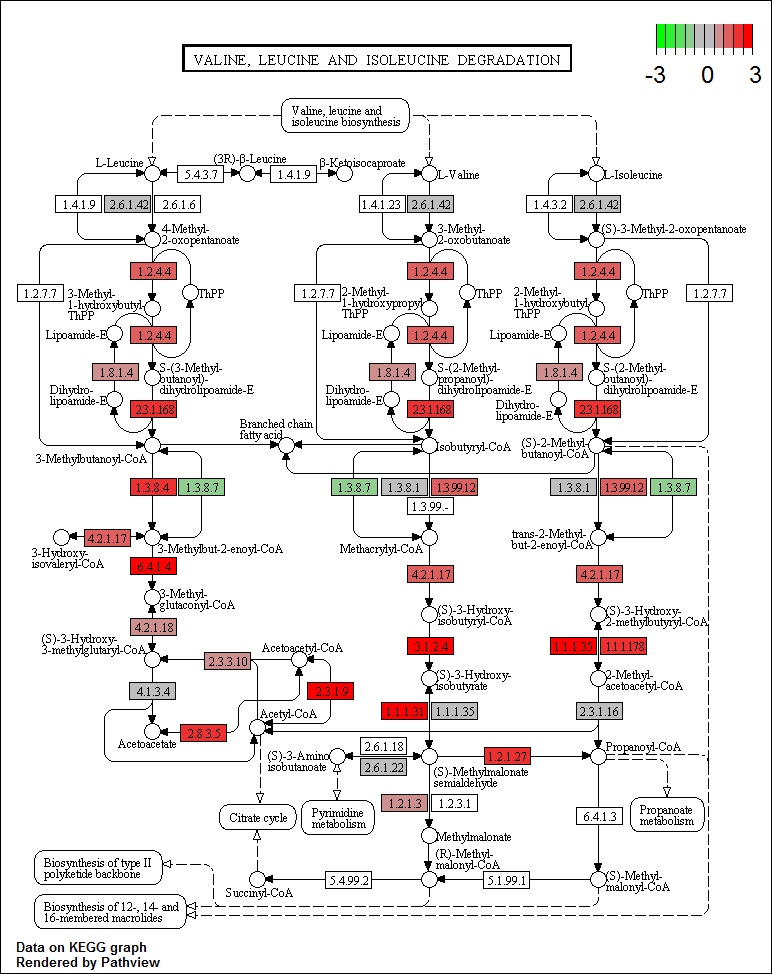

Supplement: Additional file 9: Figure S8. — Annotated KEGG map for leu, val, ile following BM. [file 13071_2015_863_MOESM9_ESM.png]

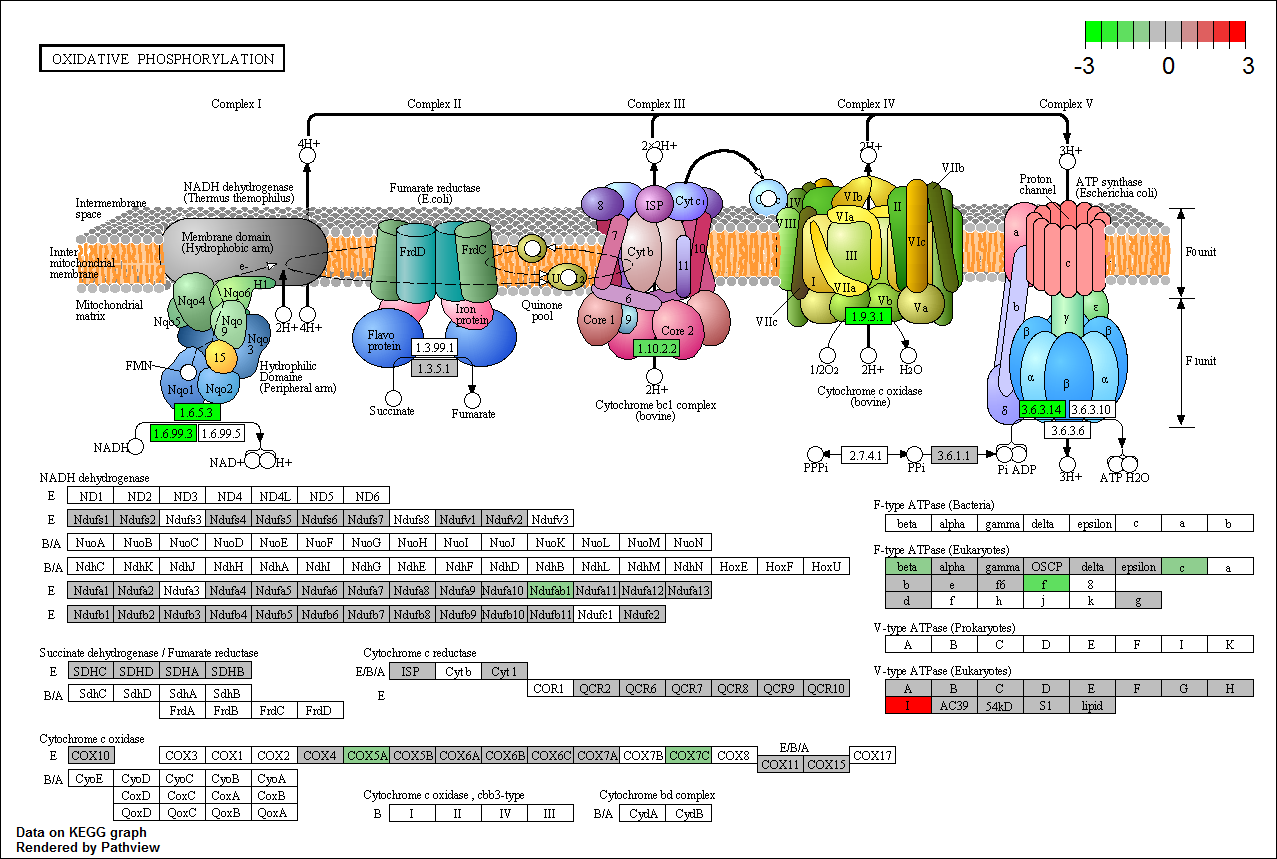

Supplement: Additional file 10: Figure S9 — Annotated KEGG map for oxidative phosphorylation, NBF. [file 13071_2015_863_MOESM10_ESM.png]

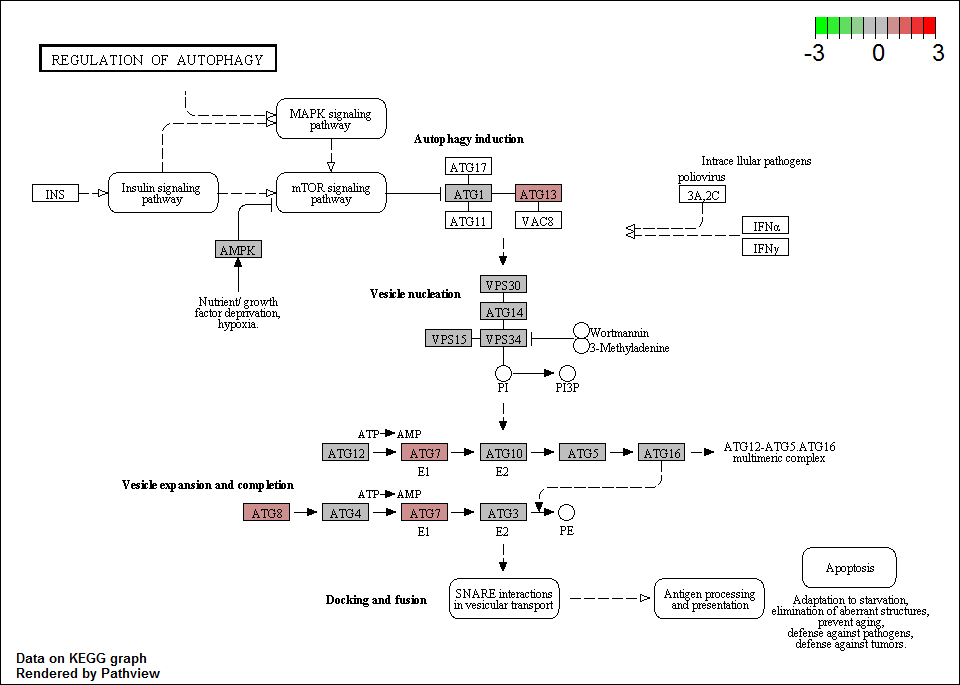

Supplement: Additional file 11: Figure S10. — Annotated KEGG map for Autophagy, NBF. [file 13071_2015_863_MOESM11_ESM.png]

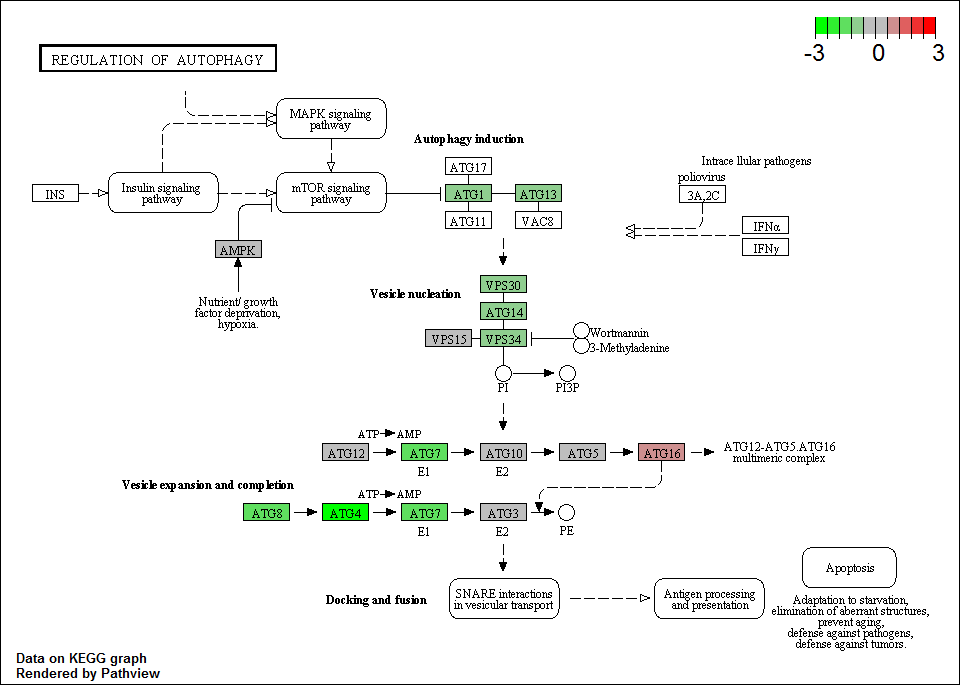

Supplement: Additional file 12: Figure S11. — Annotated KEGG map for Autophagy, following BM. [file 13071_2015_863_MOESM12_ESM.png]

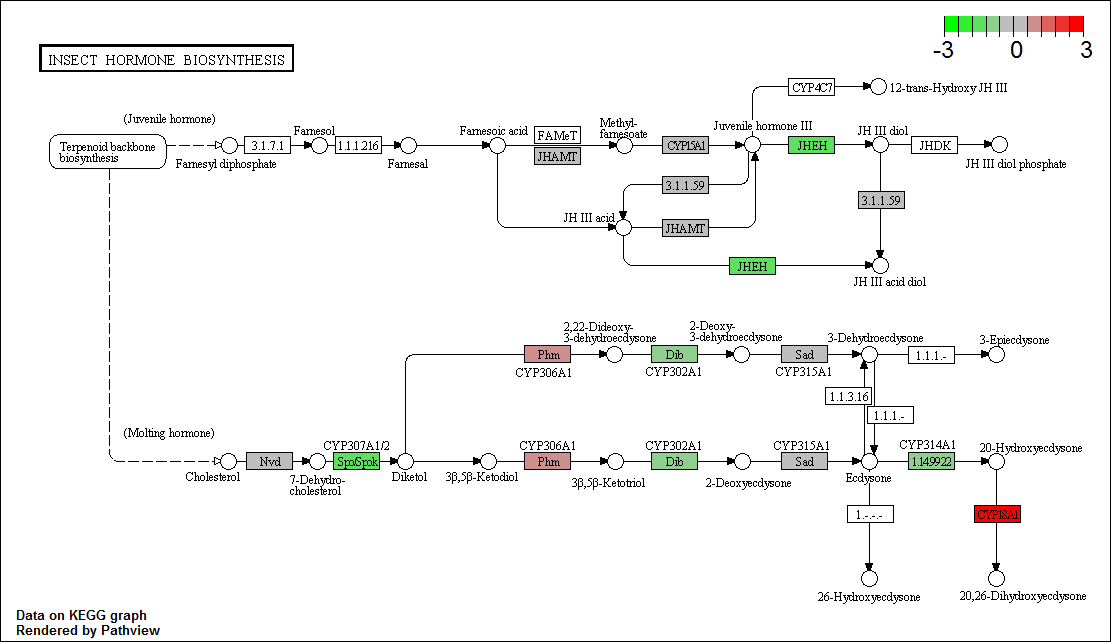

Supplement: Additional file 14: Figure S12. — Annotated KEGG map for insect hormone biosynthesis, NBF. [file 13071_2015_863_MOESM14_ESM.png]
